# Datziinae as a new subfamily name for the unavailable name Protopsychodinae Stebner et al., 2015, (Diptera: Psychodidae)

**DOI:** 10.7717/peerj.1423

**Published:** 2015-11-24

**Authors:** Frauke Stebner, Mónica M. Solórzano Kraemer, Sergio Ibáñez-Bernal, Rüdiger Wagner

**Affiliations:** 1 Steinmann-Institut, Abteilung Paläontologie, Bonn, Germany; 2 Senckenberg Forschungsinstitut und Naturmuseum, Frankfurt am Main, Germany; 3 Red Ambiente y Sustentabilidad, Instituto de Ecología, A.C., Xalapa, Veracruz, Mexico; 4 FB 10 Naturwissenschaften, Institut für Biologie, Kassel, Germany

**Keywords:** Protopsychodinae, *Datzia*, Nomenclature, Subfamily, Psychodidae

## Abstract

In a recent paper a new subfamily of Psychodidae was inadequately named Protopsychodinae. This nomenclatural act cannot be considered as a valid name under ICZN regulations because the subfamily name is not based on the type genus *Datzia* Stebner et al., 2015, and furthermore the fossil genus *Protopsychoda* Azar et al., 1999 was originally described under the subfamily Psychodinae. Therefore, the new family-group name Datziinae is herein proposed.

## Introduction

Stebner et al. (2015) recently described a new subfamily of fossil Psychodidae. However, the establishment of a new subfamily Protopsychodinae Stebner et al., 2015 with the type genus being *Datzia* is invalid under ICZN rules, article 29.1, because the subfamily name must be formed using the stem of the type genus name. Furthermore, *Protopsychoda* Azar et al., 1999 is a valid genus described within the subfamily Psychodinae which would make Protopsychodinae (if validly established with *Protopsychoda* as the type genus) a junior synonym of Psychodinae (ICZN, article 23). The name Protopsychodinae is unavailable, and instead the new subfamily name Datziinae is established, based on *Datzia* Stebner et al., 2015 as the type genus. Accordingly, the new subfamily name is herein proposed to include the type genus *Datzia* and the genus *Mandalayia* Stebner et al., 2015.

## Material and Methods

The electronic version of this article in Portable Document Format (PDF) will represent a published work according to the International Commission on Zoological Nomenclature (ICZN), and hence the new names contained in the electronic version are effectively published under that Code from the electronic edition alone. This published work and the nomenclatural acts it contains have been registered in ZooBank, the online registration system for the ICZN. The ZooBank LSIDs (Life Science Identifiers) can be resolved and the associated information viewed through any standard web browser by appending the LSID to the prefix “http://zoobank.org/”. The LSID for this publication is: urn:lsid:zoobank.org:pub:67C1C754-CFB9-45DE-AD72-62F11071AD43. The online version of this work is archived and available from the following digital repositories: PeerJ, PubMed Central and CLOCKSS.

## Systematic Palaeontology

**Table:** 

Order: Diptera Linnaeus, 1758
Family: Psychodidae Newman, 1834

Datziinae new subfamily (replacing Protopsychodinae Stebner et al., 2015, unavailable under the ICZN Code)

urn:lsid:zoobank.org:act:2C47D449-A108-401B-829E-A67DD15FB56F.

*Type-genus*: *Datzia* Stebner et al., 2015

urn:lsid:zoobank.org:act:A8D8B8AE-229E-469F-A8F8-CA496B45FFA0.

*Type-species*: *Datzia* bispina

urn:lsid:zoobank.org:act:B094011C-331E-487E-8700-B4FB53EEB95C.

*Material*: Holotype male SMF Be 2379 and 7 male Paratypes; SMF Be 2376 Paratypes 6 males. Deposited at the Senckenberg Forschungsinstitut und Naturmuseum, Frankfurt, Germany.

*Type-locality*: Hukawng Valley of the northern state of Kachin.

*Stratigraphic horizon*: 98.79 ± 0.62 million years, Upper Cretaceous (earliest Cenomanian) (Shi et al., 2012).

*Diagnosis*: unchanged (see Stebner et al., 2015, p. 6)

## Additional Information and Declarations

### New Species Registration

The following information was supplied regarding the registration of a newly described species: 1. This published work and the nomenclatural acts it contains have been registered in ZooBank. The LSID for this publication is: urn:lsid:zoobank.org:pub:67C1C754-CFB9-45DE-AD72-62F11071AD43. 2. New subfamily Datziinae: urn:lsid:zoobank.org:act:2C47D449-A108-401B-829E-A67DD15FB56F
